# An adaptive detection model for IPv6 extension header threats based on deterministic decision automaton

**DOI:** 10.1038/s41598-024-59913-8

**Published:** 2024-04-25

**Authors:** Bin Lin, Liancheng Zhang, Hongtao Zhang, Yi Guo, Shaowei Ge, Yakai Fang, Mingyue Ren

**Affiliations:** 1 School of Cyber Science and Engineering, Zhengzhou University, Zhengzhou, 450001 China; 2 National Digital Switching System Engineering and Technological Research Center, Zhengzhou, 450002 China; 3 Network Management Center, Zhengzhou University, Zhengzhou, 450001 China

**Keywords:** Electrical and electronic engineering, Computer science, Information technology

## Abstract

The IPv6 extension header mechanism, a new feature of the IPv6 protocol, enhances flexibility and scalability but introduces numerous security threats like firewall evasion and covert channels. Existing threat detection methods face limitations in detection types, universality, and speed. Hence, an adaptive detection model for IPv6 extension header threats (ADM-DDA6) is proposed. Firstly, standard rule sets are designed for common IPv6 extension headers, successfully detecting 70 types of threats from THC-IPv6 and ExtHdr tools using only 20 rules. Secondly, by parsing IPv6 extension headers, matching rules, establishing transition relationships, and deciding packet threat status based on final states (Normal or Abnormal), complex threats like header disorder and header repetition can be detected. Finally, an adaptive rule matching method is introduced, which dynamically selects rule sets based on IPv6 extension header types, effectively reducing rule matching time. Experimental results show that under different threat magnitudes, ADM-DDA6 is 32% faster than Suricata v6.0.12 and 21.2% faster than Snort v3.1.61.0 in detection speed. Additionally, as the number of threats increases, on commodity hardware, ADM-DDA6 incurs only a 0.7% increase in CPU overhead with no significant memory consumption increase, maintains maximum throughput, and exhibits minor performance changes under low and moderate network load conditions.

## Introduction

The widespread implementation and utilization of IPv6 have evolved into an irreversible trend^1^. The IPv6 extension header mechanism is a standout feature introduced by the IPv6 protocol, providing flexible support for network services. These extension headers enable the inclusion of extra information in packets, allowing the application of specific functionalities to IPv6 packets, and providing a broader range of choices and flexibility for various services.

However, the use of IPv6 extension headers is suboptimal, with a noticeable occurrence of packet loss. According to information from the APNIC (Asia Pacific Network Information Center) on a global scale, there is a notable packet loss rate for IPv6 extension header packets (November 1–7, 2023)^2^. The loss rates for packets with Fragment headers and Hop-by-Hop (HBH) Options headers are 21.72% and 33.02%, respectively. Remarkably, packets with Destination Options headers (DOH) experience an exceptionally high loss rate of 99.75%.

Many studies have examined the actual use of IPv6 extension headers in networks, showing that while usage has increased with the wider adoption of IPv6, their overall utilization rate remains low^3–9^. This finding is consistent with recent data from APNIC. Recent research suggests that the security threats within IPv6 extension headers are one of the contributing factors to this issue^10^. So far, a practical solution to address these IPv6 extension header threats is still lacking, leading to the need to discard IPv6 packets carrying extension headers directly^11^.

The limited utilization of IPv6 extension headers indicates that some of their functionalities and features remain underutilized. This not only poses a potential challenge to the integrity and scalability of the IPv6 protocol but also serves to impede the widespread deployment of IPv6 on a global scale to some extent^12^. The research conducted by Hamarsheh et al. sheds light on the deployment challenges of IPv6, highlighting additional significant issues encountered during the transition process^13^.

Consequently, this study focuses on exploring a detection scheme aimed at addressing threats associated with IPv6 extension headers. The objective is to enhance the security and reliability of IPv6 extension headers, thereby facilitating the application and realization of the advantages offered by IPv6 deployment.

The main source of threats within IPv6 extension headers arises from their misuse, involving the malicious crafting of packets that do not comply with RFC specifications to initiate threats^14^. In 2012, Atlasis used this method to create IPv6 extension header packets with various threats, generating a multitude of identical or diverse header types and transmitting arbitrary data within HBH headers^15^. Additionally, Atlasis explored vulnerabilities in IPv6 Fragment headers, creating additional threat vectors based on these headers, including tiny fragments or overlapping fragments^16,17^. Testing on various mainstream operating systems (OS) and intrusion detection systems (IDS) revealed that many OS implementations did not fully comply with the specifications, and IDSs faced challenges in detecting these threats.

Misusing IPv6 extension headers can facilitate the evasion of threats that evade security systems, allowing actions like port scanning and SQL (Structured Query Language) injection on a target without triggering alarms^18–23^. Adversaries can also exploit these vulnerabilities for activities like OS fingerprinting, covert channels, and denial of service (DoS) threats^24–26^. Therefore, there is an urgent need to establish robust protection methods for IPv6 extension header threats.

Currently, protection methods against threats within IPv6 extension headers can be categorized into three main types: First, due to the potential security threats posed by IPv6 extension headers, devices are sometimes configured to discard all such traffic. Although effective in mitigating threats, severely impedes the legitimate use of IPv6 extension header traffic^27^. Second, threat detection based on deep packet inspection (DPI), while capable of effectively identifying certain extension header threats, suffers from limited coverage of threat types. Additionally, its specific implementation relies on particular protocols or devices, lacking universality^28–30^. Third, IDSs are based on rule matching, such as Suricata^31^ and Snort^32^.

The prior works indicate that Suricata and Snort are currently the most supportive IDSs for IPv6^20,26^. However, both two IDSs have a limited number of rules addressing threats within IPv6 extension headers, providing coverage for only a narrow range of threat types.

**Figure 1:**
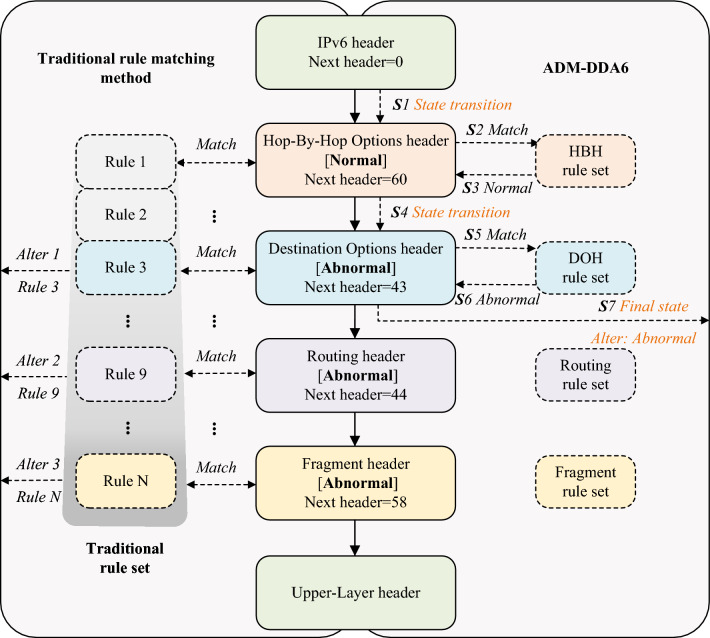
The illustration demonstrates the detection process of an IPv6 extension header packet (center), comparing the workflow between the traditional rule matching method (left) and ADM-DDA6 (right). The IPv6 extension header packet (center) contains the IPv6 header, a normal HBH header, an abnormal DOH header, an abnormal Routing header, a normal Fragment header, and the upper-layer header. Headers are identified through the next header field in the preceding header, connected by arrows. This paper establishes standard rule sets for common extension headers based on RFC specifications, respectively. On the right, rules include the HBH, DOH, Routing, and Fragment rule sets respectively. Taking steps S1-S7 as an example, different headers are parsed with the ADM-DDA6 model, enabling state transfers between headers (i.e., S1, S4, and S7). An adaptive rule matching method is proposed for each header parsing process, aiming to reduce matching attempts by adaptively matching the current extension header with the corresponding rule set (i.e., S2, S3, S5, S6) and terminates the entire detection process upon anomaly detection (i.e., S6, S7).

Additionally, traditional rule matching methods may require traversing the entire rule^33^, and there are cases where many rules are matched for a single packet(e.g., the left side of Fig. 1). To alleviate the problems of traditional rule matching methods, which include the lack of extension header rules resulting in limited threat detection types and multiple matching attempts during rule matching, this paper focuses on designing more comprehensive rules and reducing the number of rule matching.

Furthermore, this paper leverages the advantages of the deterministic finite automaton (DFA) theory, which describes a finite number of states and transitions between states, we use DFA theory for rule matching and threat-detecting processes related to a finite number of IPv6 extension header types.

This paper introduces an adaptive detection model for IPv6 extension header threats based on deterministic decision automaton (ADM-DDA6, as indicated on the right side of Fig. 1). By defining a set of states to represent different header types and detection states, constructing a state transition function, and parsing IPv6 extension headers while simultaneously matching rules, the results of header parsing and rule matching provide deterministic state transition relationships. Subsequently, the current state is assessed. If it is a final state (i.e., S7), a decision regarding the threat status of the packet is made based on whether the state is normal or abnormal.

The main contributions of this paper include four aspects.

1. **Formulation of standard RFC-compliant rule sets for common IPv6 extension headers (Section “Standard extension header rules formulation based on RFC specifications”).** By complying with RFC specifications, this approach enables the detection of a broader range of threat types with a minimal and more standardized set of rules. Using the example of IPv6 fragment evasion threats, the paper details the rule formulation process based on RFC 7112 (Section “Formulating rules based on RFC 7112”), and provides a clear rule table (Section “Overview of rule formulation of IPv6 extension headers”) for common extension headers to significantly enhance threat detection capabilities.
2. **Proposal of the ADM-DDA6 model (Section “Problem analysis and proposed model”)**. This paper proposes the ADM-DDA6 model to simulate the threat detection process for complex and dynamic IPv6 extension headers. This model employs 8 header states (such as IPv6 header, HBH header, DOH header, etc.) and 2 terminal states (Normal and Abnormal) to model IPv6 extension header threat detection (Fig. 5). The transition conditions are based on the next header field of the extension header and the results of rule matching against the current header, providing a universal detection model.
3. **Proposal of an adaptive rule matching method for ADM-DDA6 (Section “An adaptive approach for rule matching”).** This paper presents an adaptive rule matching method for the ADM-DDA6 detection process (Fig. 6). Whenever ADM-DDA6 parses an extension header, it adaptively selects the rule set corresponding to the current header type for matching. This approach ensures effective detection while reducing the number of rule matches, thereby enhancing threat detection speed.
4. **Implementation and evaluation of ADM-DDA6 (Section “Experimental comparison and analysis”).** This paper implements the ADM-DDA6 adaptive detection method for IPv6 extension header threats, creating a test suite (named **ExtHdr**) with a more even distribution of threat types, containing 40 types of IPv6 extension header threats to evaluate ADM-DDA6’s detection capabilities. Experimental evaluations cover threat detection types, detection times, and resource overhead. The results demonstrate that ADM-DDA6 indeed fulfills the characteristics of diverse detection types, universal detection methods, and fast detection speed, with low performance overhead.

The remainder of this paper is organized as follows. The second section discusses the related work concerning the IPv6 extension header threats. The third section introduces the formulation of standard RFC-compliant rule sets for common IPv6 extension headers. In the fourth section, the ADM-DDA6 model is explained, and in the fifth section, the protection capability of ADM-DDA6 is evaluated from different aspects. Finally, the sixth section concludes the paper.

## Related work

This section focuses on the research status of IPv6 extension header threats and IPv6 extension header threat protection.

### Research status of IPv6 extension header threats

In the 2012 Black Hat conference, Atlasis found vulnerabilities in IPv6 extension headers using the Scapy tool. He created threat packets that did not comply with RFC 2460 specifications^15^. The threats mainly included using many identical or different extension headers and exploiting vulnerabilities like sending arbitrary data in HBH or DOH headers. Subsequently, Atlasis explored vulnerabilities in the IPv6 Fragment header, creating several threats based on Fragment headers. These vulnerabilities include tactics like tiny fragments (fragment lengths less than 1280 bytes), overlapping fragments, and atomic fragments^16,17^. Atlasis checked how well various popular OSs (Windows, Linux, BSD, etc.) and IDSs handled these threats. The results indicated that many OS’s protocol stacks did not comply with RFC specifications. Also, both Snort and Suricata of that time couldn’t identify or counter these threats.

The misuse of extension headers can also create evasion threats that evade security systems. Numerous studies have tested this kind of threats, showing that such threats can indeed evade detection by specific security systems, including firewalls^19^, IDS like Suricata and Snort^28^, ACLs (Access Control Lists) on routers and switches^19^, and commercial intrusion detection and prevention system (IDPS) devices such as HP Tipping Point and Sourcefire^20–22^. Despite the widespread use of IPv6, several studies indicate that these vulnerabilities persist^23^. Adversaries can take advantage of these vulnerabilities for activities such as OS fingerprinting, covert channels, and DoS threats^18,34^.

### Research status of IPv6 extension header threat protection

Current protection methods against threats posed by IPv6 extension headers can be categorized into three main types.

1. Direct discard of all IPv6 extension header traffic
  The proposed approach involves configuring IPv6 nodes to directly discard IPv6 extension header packets such as DOH headers and HBH headers^18,34^. While the advantage of this approach lies in its simplicity and directness, allowing interception of all potential threats, its primary drawback resides in its tendency towards excessive absolutism when filtering IPv6 extension header packets. This may lead to the inadvertent filtering of legitimate packets, resulting in a higher rate of false positives and subsequent issues with connection or deployment failures^27^. Recent studies have further indicated that a significant portion of IPv6 extension header traffic is benign and necessary, thus advocating against its wholesale disposal^6,35^.
2. Threat detection based on deep packet inspection
  DPI is an advanced traffic classification technique that enables a detailed analysis of received traffic. Tajdini M proposed a detection scheme named NOPO for IPv6 DoS threats, but this scheme is limited to detecting only 6 types of IPv6 extension header threats^28^. Al-Ani et al. utilized OpenFlow 1.3.0 features to detect IPv6 extension headers in an OpenFlow network, presenting the ofsoftswitch scheme to detect maliciously bypassing OpenFlow firewalls with fragmented packets^29^. However, the implementation of this approach depends on the OpenFlow protocol. Naagas et al. introduced a new security model, DEH-DoSv6, which adds a traffic classification layer between the router forwarding plane and the control plane to filter malicious packets^30^. Nevertheless, the implementation of this scheme relies on router devices. Consequently, such methods may not cover a comprehensive range of threat types and lack universality.
3. Intrusion detection systems based on rule matching
  Suricata and Snort, as two prominent open-source IDSs with comprehensive support for IPv6^20^, leverage extensive rule sets to cover a multitude of potential threat types, including malicious extension header traffic^36^, thereby enhancing the breadth of threat detection. Despite the favor garnered by rule matching methods due to their versatility and widespread adoption (such as in IDSs and firewalls), there still exist challenges concerning the limited variety of threat detection for IPv6 extension headers and the relatively slow detection speed.

In summary, the current protective measures suffer from limitations in detection variety, poor generality, and suboptimal detection speed. Recognizing the significant role of rule matching methods in threat prevention, this study draws inspiration and proposes an ADM-DDA6 method based on DFA theory. In contrast to traditional rule matching methods, this approach offers several innovative features:

- Establishing RFC-compliant rule sets for IPv6 extension headers, thereby reducing the number of rules while expanding the scope of threat detection types;
- Introducing the ADM-DDA6 model based on DFA theory to simulate the parsing and detection process of IPv6 extension header packets, capable of addressing complex and dynamic combinations of IPv6 extension headers;
- Proposing an adaptive rule matching method for the detection process of ADM-DDA6, wherein rules are matched only against the current header, and the detection process is promptly terminated upon encountering any anomalies during header parsing.

In essence, this approach draws upon the principles of rule matching. Leveraging this framework, novel rule sets for IPv6 extension header threats, a new threat detection model, and an innovative rule matching method have been devised.

## Standard extension header rules formulation based on RFC specifications

To implement ADM-DDA6, it is essential to establish rule sets for common IPv6 extension headers. This section begins by analyzing the advantageous characteristics of formulating rules for IPv6 extension headers based on RFC specifications and subsequently, taking the IPv6 fragment evasion threats as an example, formulating rules for such threats. Finally, a comprehensive table of rules formulated for each type of extension header is presented.

### Analyzing the advantageous characteristics of standard rules

Based on the summary of related work^15,22^, this paper observes that threats from IPv6 extension headers primarily stem from the misuse of RFC specifications, which involves constructing packets that do not comply with the standards. RFC 9099 also indicates that the reception or forwarding of such non-compliant packets by nodes can lead to node crashes, mandating the discarding of all non-compliant IPv6 packets according to RFC specifications^37^. Based on these findings, the paper concludes that all non-compliant IPv6 extension header packets are considered abnormal. The advantages of the proposed standard RFC-compliant rule sets are as follows:

- **Increased detection of threat types. **Non-compliant packets are inherently abnormal, allowing for the detection of a wider range of threat types, including some zero-day threats. This method is effective because emerging threats often exploit vulnerabilities of network protocol specifications^38^.
- **Reduced rules numbers. **As RFC specifications are finite, the number of rules formulated based on these specifications is inherently finite. This helps simplify the rule set and improves the speed of rule matching.
- **Improved rule matching speed.** Traditional IDS rule set often contains a large number of rules, leading to situations where a single packet may match multiple rules, thereby slowing down rule matching. The adaptive rule matching method proposed in this paper only requires matching with the rule set corresponding to the current IPv6 extension header. With fewer rules, this approach enhances the speed of rule matching.

### Formulating rules based on RFC 7112 (An example of IPv6 fragment evasion threats)

The threat of IPv6 fragment evasion is a common threat method that exploits vulnerabilities in the IPv6 fragmentation mechanism to evade detection by security systems such as firewalls.

According to RFC 7112 specifications^39^, the first fragment must contain the complete upper-layer protocol header. However, the IPv6 fragment evasion threat scatters the complete upper-layer protocol header across different fragments, thereby evading firewall detection^40^.

**Figure 2:**
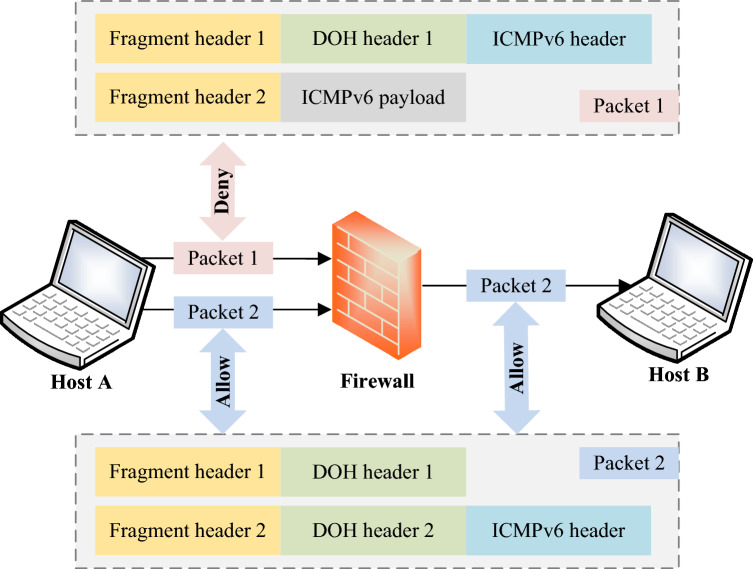
An example of the IPv6 fragment evasion threats.

Taking Fig. 2 as an example, the firewall blocks ICMPv6 (Internet control message protocol version 6) echo request messages from any host to Host B. Therefore, the fragmented packet 1 sent by Host A is detected and denied by the firewall. However, the adversary utilizes the fragmentation mechanism to place the ICMPv6 header in the second fragment, constructing packet 2. This causes the firewall to be unable to detect the ICMPv6 echo request header, allowing packet 2 to successfully evade the firewall. Thus, the fragment evasion threat occurs^16,29^.

In fact, due to the high flexibility and complexity introduced by IPv6 extension headers, the types of fragment evasion threats are more diverse and intricate. However, their principles are similar to those illustrated in Fig. 2, involving making the first fragment lack complete upper-layer header information or even omitting upper-layer header information entirely. RFC 7112 refers to this behavior as an “incomplete IPv6 header chain”^39^.

IPv6 fragment evasion threats can modify the upper-layer protocol header to any other protocol type to suit their threat objectives. For example, by constructing fragmented packets with an upper-layer protocol set as TCP (Transmission Control Protocol) and a destination port as 80, adversaries can create XSS (Cross-Site Scripting) threats. Firewalls are unable to detect and protect against such threats^22^.

**Figure 3:**
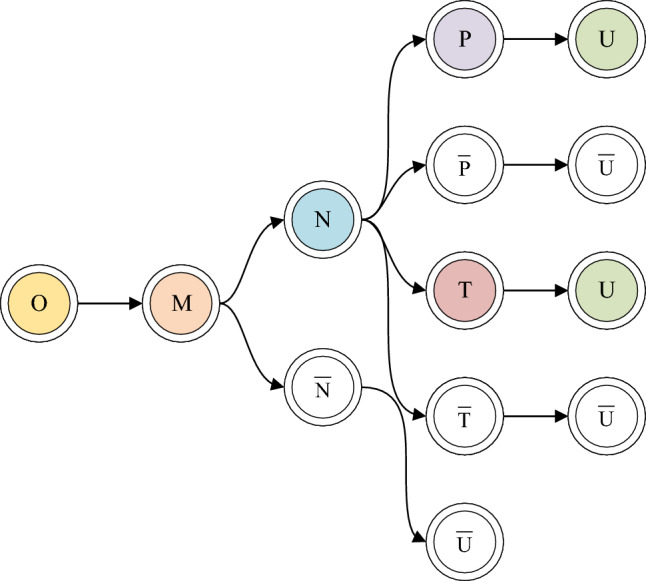
Logical graph of rules generation.

Analyzing the characteristics of the IPv6 fragment evasion threat, this paper proposes a logical diagram for rule generation, as depicted in Fig. 3.

*U*: Signifies a complete IPv6 header chain, complying with RFC 7112.

*O*: Represents the Offset field value of the IPv6 Fragment extension header being 0.

*M*: Denotes that the More Fragments field value of the IPv6 Fragment extension header is 1.

*N*: Indicates that the Last Next Header (LastNH) of the last extension header in the first fragmented packet is equal to the protocol type of the upper-layer protocol header.

*P*: Signifies that the upper-layer protocol header length in the first fragmented packet is greater than or equal to 8 bytes.

*T*: Represents that the upper-layer protocol header length in the first fragmented packet is greater than or equal to 20 bytes.

As shown in Fig. 3, it is evident that obtaining *U* can occur through two paths, $O\rightarrow M\rightarrow N\rightarrow P\rightarrow U$ and $O\rightarrow M\rightarrow N\rightarrow T\rightarrow U$. Abstracting these two paths into two rule expressions, each rule corresponds to a specific scenario. Finally, combining these rules yields a singular rule, denoted as ${{R}_{U}}$ that aligns with the specifications outlined in RFC 7112:

1 $$\begin{aligned}{} & {} {{R}_{U1}}=\left( O\wedge M\wedge N\wedge P \right) \end{aligned}$$

2 $$\begin{aligned}{} & {} {{R}_{U2}}=\left( O\wedge M\wedge N\wedge T \right) \end{aligned}$$

3 $$\begin{aligned}{} & {} {{R}_{U}}=\left( O\wedge M\wedge N\wedge P \right) \vee \left( O\wedge M\wedge N\wedge T \right) \end{aligned}$$

The combination of *O* and *M* indicates that the current fragment packet is the first fragment. Without the constraint of the *M* condition, there is a possibility of obtaining an atomic fragment packet. However, such a packet is not enough to constitute a fragment evasion threat.

When *N* = True, it signifies knowledge of the upper-layer protocol type. Taking common upper-layer protocols as examples, when LastNH = 6, it implies that the upper-layer protocol is TCP, and when LastNH = 58, it signifies that the upper-layer protocol is ICMPv6. When *N* = False, it indicates an unknown upper-layer protocol type. For instance, if LastNH = 60, the next header is a DOH header rather than an upper-layer protocol header. Thus, when *N* = False, it unequivocally suggests the occurrence of a fragment evasion threat.

Common upper-layer protocol types include TCP (6), UDP (17), ICMPv6 (58), and others. Given that the header lengths of ICMPv6, UDP (User Datagram Protocol) are 8 bytes, when *P* = True, it indicates that the first fragment contains a complete upper-layer protocol header, constituting a normal packet. Conversely, when *P* = False, it suggests incompleteness in the upper-layer protocol headers, particularly for ICMPv6 and UDP, signifying the occurrence of an IPv6 fragment evasion threat. Rule 2 is specific to the upper-layer protocol type TCP. As critical information for TCP resides within the first 20 bytes, when *T* = True, it signifies the integrity of the TCP header.

In summary, we have formulated a rule for the IPv6 Fragment header, aimed at detecting whether the first fragmented packet complies with this rule. If the first fragmented packet fails to meet this rule, we can infer that the packet poses a fragment evasion threat.

### Overview of rule formulation of IPv6 extension headers

The IPv6 extension headers serve as a useful and optional addition to IPv6 functionality. It is worth noting that IPv6 packets are not obligated to include extension headers. Therefore, the incorporation of extension headers can be utilized to improve the flexibility and scalability of IPv6.

Table 1 offers a concise description of IPv6 extension headers, including the Hop-by-Hop (HBH) Options header, Destination Options header (DOH), Routing header (Routing), Fragment header (Fragment), Authentication header (AH), and Encapsulation Security Payload (ESP) header.

**Table 1:** Types and descriptions of IPv6 extension headers.

Extension header	Next header	Description
HBH	0	Read by devices in the transit network
DOH	60	Read by destination devices
Routing	43	Contains methods to support making a routing decision
Fragment	44	Contains parameters of packet fragmentation
AH	51	Information regarding authenticity
ESP	50	Encryption information

**Figure 4:**
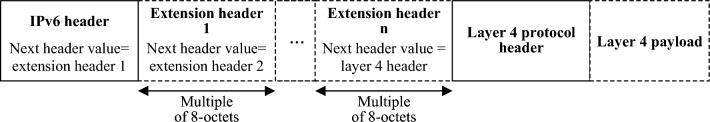
IPv6 packet structure.

An IPv6 packet can contain zero, one, or multiple extension headers. These optional extension headers separate the IPv6 header and the upper-layer header (e.g., TCP header), with each extension header identified by the Next Header (NH) value in the preceding header^41^ (as shown in Fig. 4). Other extension headers may only appear once in the packet, except for the DOH header. Figure 4 illustrates the general structure of an IPv6 packet, positioning these headers between the IPv6 header and the upper-layer header in the packet.

This paper establishes rule sets for common types of IPv6 extension headers, as outlined in Table 2. These rules ensure compliance and security in header communication, mitigating potential risks and abnormal behavior.

**Table 2:** Rule descriptions of IPv6 extension headers.

Extension header	Rule description
HBH	R1: Payload Length must be 0, Next Header must be HBH header, and Jumbo Payload length must be greater than or equal to 65,535^42^.
	R2: Can appear at most once and must appear after the IPv6 header^41^.
	R3: PadN Option Data consists of all zero-valued octets^41^.
	R4: Deny unknown Option Type^41^.
DOH	R5: Must appear at most twice^41^.
	R6: PadN Option Data consists of all zero-valued octets^41^.
	R7: Deny unknown Option Type^41^.
Routing	R8: Routing Type must not be equal to 0^43^.
	R9: Routing Type must be 2 and Segments Left must be 1^44^.
	R10: Can appear at most once^41^.
Fragment	R11: Res field must be 0^41^.
	R12: MTU must be greater than or equal to 1280 bytes^41,45^.
	R13: Deny overlapping fragments^46^.
	R14: Deny atomic fragments^47^.
	R15: Deny nested fragments^41^.
	R16: The payload length field of the reassembled packet must be less than 65,536 bytes^41^.
	R17: Each fragment, except possibly the last (“rightmost”) one, is an integer multiple of 8 octets long^41^.
	**R18: Completed IPv6 header chain** ^39^.
AH	R19: Res field must be 0^41^.
	R20: Can appear at most once^41^.

Significant values are in bold.

Considering that ESP plays a pivotal role in IPsec as a component with robust security features, and given the current absence of research focusing on threats specifically targeting ESP headers, an extensive discussion on dedicated protective measures for ESP might not be immediately imperative. However, it is crucial to maintain vigilance and continuously evaluate the necessity of such rules in response to evolving threat landscapes.

Using the example of the IPv6 fragment evasion threat, Rule 18 in the fragment rule set effectively defends against such malicious behaviors. Specifically, Rule 18 rejects all packets with incomplete IPv6 header chains, affirming the rationality and effectiveness of the formulated rules.

## ADM-DDA6 model

Based on the rule sets, ADM-DDA6 can perform threat detection for IPv6 extension headers. Nevertheless, current detection methods still suffer from poor universality and slow detection speeds. In this section, a universal DFA model is introduced to simulate the threat detection process for IPv6 extension headers. Furthermore, an adaptive rule matching method is proposed to expedite the speed of threat detection.

### Problem analysis and proposed model

#### Problem analysis

IPv6 extension headers have many types and variable lengths, which give IPv6 packets a variety of structures and functions. The misuse IPv6 of extension headers can pose a series of challenges for threat detection.

RFC 9099 summarizes this misuse into two main categories: disorder and repetition of headers that do not conform to the RFC specifications^37^. In this context, this paper employs IPv6 header (A), HBH header (B), DOH header (C), Routing header (D), and Fragment header (E) to construct nine combinations of IPv6 header chains (as shown in Table 3). These combinations include normal combinations, disorderly combinations, repeated combinations, and combinations of disorder and repetition, representing 5 types of scenarios.

**Table 3:** Complex and variable IPv6 header chains.

IPv6 header chain types	IPv6 header chains
Normal	A+B+C+D+E
Header disorder	A+C+B+D+E
	A+B+D+C+E
	A+D+E+B+C
Header repetition	A+B+B+C+D+E
	A+B+C+C+D+E
	A+B+C+D+D+E
Header disorder + repetition	A+C+B+B+D+E
Header repetition + disorder	A+B+B+D+C+E

Based on these scenarios, adversaries can create numerous, more complex threat packets based on IPv6 extension headers. The presentation of these packets is dynamic and intricate, lacking a fixed pattern, which poses significant challenges to threat detection^37^.

Currently, a universal solution for the threat detection of IPv6 extension headers is lacking. To fill this gap, this paper proposes the ADM-DDA6 model to simulate the threat detection process for IPv6 extension header packets.

#### ADM-DDA6 model

DFA is a mathematical model used to describe systems with finite states and transitions between these states. In a DFA, the system, based on input and its current state, can uniquely determine the state relationship and transition to the next state through state transitions^48^.

**Figure 5:**
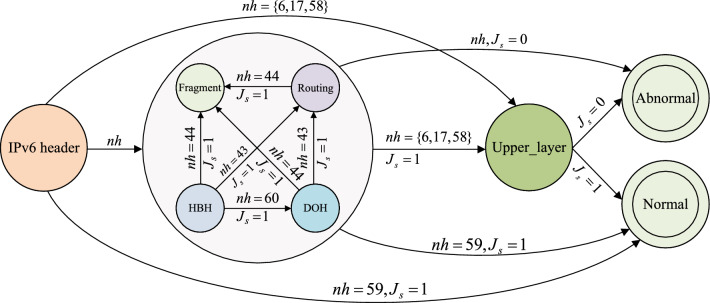
ADM-DDA6 model.

Common IPv6 extension headers consist of only six types (as shown in Table 1). A DFA is well-suited to adapt to this type of finite and structurally complex IPv6 header chain. This paper abstracts the ADM-DDA6 model as a finite automaton M (as shown in Fig. 5). M has 8 states, denoted as IPv6 header, HBH, DOH, Routing, Frag, Upper_layer, Normal, and Abnormal. IPv6 header is the start state, Normal and Abnormal are final states denoted with double circles, while the remaining states represent the header states in the threat detection process. Arrows pointing from one state to another represent transitions.

The formal definition of the rule matching process is as follows:

4 $$\begin{aligned}{} & {} M=\{Q,I,\delta ,{{q}_{0}} ,F\} \end{aligned}$$

Among:

*Q* is a set of states, with each element referred to as a state:

5 $$\begin{aligned}{} & {} Q = \{IPv6\text { }header,HBH,DOH,Routing,Fragment,Upper\_layer,Normal,Abnormal\} \end{aligned}$$

*I* is an input set, with each element referred to as an input, $I=\left\{ nh,{{J}_{s}} \right\}$. *nh* is the next header field in the previous header, indicating the type of the current parsed extension header. ${{J}_{s}}$ represents the result of the current parsing extension header rule set to match. When ${{J}_{s}}=1$, it indicates a successful rule match, the extension header is considered normal. When ${{J}_{s}}=0$, it indicates a rule match failure, the extension header is deemed abnormal.

$\delta :Q\times I\rightarrow Q$ is the transition function, a mapping on $Q\times I$, i.e., $\delta \left( {{Q}_{i}},a \right) ={{Q}_{i+1}},\left( {{Q}_{i}},{{Q}_{i+1}}\in Q,a\in I \right)$. For example, $\delta \left( HBH,\left( nh=60,{{J}_{s}}=1 \right) \right) =DOH$ signifies that after parsing the HBH header, the result is that this input indicates that the next extension header to be parsed is the DOH header (i.e., nh = 60). The detector’s rule match result for the HBH header is 1, signifying that the HBH header is normal. Consequently, the transition occurs to the parsing of the DOH header.

${{q}_{0}}\in Q$ is the start state, i.e., the first IPv6 header state to be parsed.

$F\subseteq Q$ is the final state, $F=\left\{ Normal,Abnormal \right\}$. *Normal* indicates that the entire packet parsing is completed, and it is a normal packet. *Abnormal* indicates that the currently parsed extension header is abnormal, and therefore, the entire packet is deemed abnormal.

In the ADM-DDA6, if every extension header is normal (i.e., ${{J}_{s}}=1$), then the final state of M will certainly be *Normal*. Conversely, if the result of parsing any extension header is ${{J}_{s}}=0$, there is no need to continue parsing subsequent headers. Instead, the state transitions directly to *Abnormal*. This is distinct from traditional rule-based IDS, which matches the features of a packet against all rules in its library after parsing a packet, incurring substantial detection overhead.

### An adaptive approach for rule matching

#### Problem analysis

The primary goal of this paper is to detect threats from IPv6 extension header packets and issue timely warnings. Accordingly, when ADM-DDA6 initially detects any irregularities within a specific extension header, it should promptly transition to a final state and issue a warning.

Illustratively, consider the menacing packet depicted in Fig. 1. It comprises one normal HBH header, one abnormal DOH header, one abnormal Routing header, one normal Fragment header, and an upper-layer protocol header.

Traditional rule matching methods sequentially evaluate incoming packets against a repository of N rules (depicted on the left side of Fig. 1). If the matching is successful, a warning is either directly issued based on the corresponding rule or an attempt is made to match subsequent rules. As depicted on the left side of Fig. 1, this traditional method ultimately successfully detects 3 abnormal rules and issues corresponding warnings.

To expedite the detection speed of IPv6 extension header threats, this paper leverages the distinctive characteristics of these headers. For each header type, a standardized rule set is formulated, giving rise to the proposal of an adaptive rule matching method, as depicted on the right side of Fig. 1. A detailed exposition of this process can be found in Section “Running example verification”.

#### Adaptive rule matching model

To explain the adaptive rule matching method more effectively, this paper conducts a modeling for the proposed method. It begins by outlining the behaviors associated with each extension header type. Following this, a threat detector *J* is introduced to assess whether these behaviors pose a threat. For each extension header type (nh), a corresponding rule set $R_{nh}$ is formulated, and threat detection is carried out using the threat detector *J*.

We define the set of behaviors *U*, which comprises two subsets: normal behavior (*N*) and abnormal behavior (*A*).

6 $$\begin{aligned}{} & {} N\cup A=U \end{aligned}$$

7 $$\begin{aligned}{} & {} N\cap A=\varnothing \end{aligned}$$

We model behaviors based on IPv6 extension headers using the mapping D:

8 $$\begin{aligned}{} & {} D\left( nh,U \right) =n{{h}^{U}} \end{aligned}$$

9 $$\begin{aligned}{} & {} D\left( nh,N \right) =n{{h}^{N}} \end{aligned}$$

10 $$\begin{aligned}{} & {} D\left( nh,A \right) =n{{h}^{A}} \end{aligned}$$

11 $$\begin{aligned}{} & {} n{{h}^{N}}\cup n{{h}^{A}}=n{{h}^{U}} \end{aligned}$$

We define a threat detector $J=\left( s,{{R}_{nh}} \right) ,s\in n{{h}^{U}}$, consisting of two parts: *s* is the behavior function based on IPv6 extension headers, and $R_{nh}$ is the rule set corresponding to a specific extension header (*nh*).

12 $$\begin{aligned}&J\left( s \right) =\left\{ \begin{matrix} 1 &{} s=Normal \\ 0 &{} s=Abnormal \\ \end{matrix} \right. \end{aligned}$$

**Figure 6:**
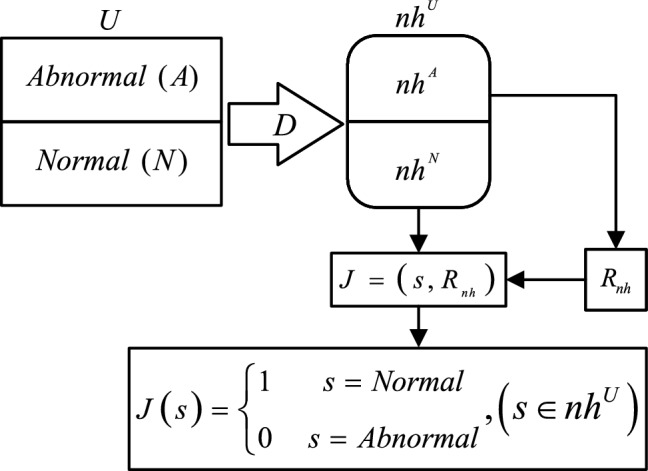
Adaptive rule matching process.

The adaptive rule matching method is illustrated in Fig. 6. First, ADM-DDA6 parses the header information. Following this, the header undergoes rule matching against the standard rule set designed for it. Eventually, the rule matching results for this extension header are obtained and returned to ADM-DDA6.

### Running example verification

In this section, the packet in Fig. 1 is taken as an example and the process of threat detection on it using ADM-DDA6 is analyzed in detail in combination with the state transition process in Fig. 5:

- S1. ADM-DDA6 parses the basic IPv6 header, and the next header field of the basic header is found to be 0 (i.e., $nh=0$). According to $nh=0$, transition to the next state, which is the parsing state for the next header (i.e., HBH header) as illustrated in Fig. 5.
- S2. ADM-DDA6 matches the rule set for the HBH header.
- S3. HBH header parsing is normal, and it returns the next header field value $nh=60$ and the result of the rule matching ${{J}_{s}}=1$.
- S4. ADM-DDA6 transitions to the next state based on $nh=60$ and ${{J}_{s}}=1$, entering the parsing state for the next header (as illustrated in Fig. 5).
- S5. ADM-DDA6 matches the rule set for the DOH header.
- S6. DOH header parsing is abnormal, and it returns the next header field value $nh=43$ and the result of the rule matching ${{J}_{s}}=0$.
- S7. ADM-DDA6 transitions to the next state based on $nh=43$ and ${{J}_{s}}=0$. According to Fig. 5, regardless of the value of *nh*, if ${{J}_{s}}=0$, the system will transition to the final state “Abnormal”. Therefore, ADM-DDA6 ultimately determines that the packet is abnormal.

In contrast to the traditional rule matching approach (left side of Fig. 1), ADM-DDA6 reduces both the number of rules that need matching and the frequency of rule matching.

## Experimental comparison and analysis

This section begins by introducing the experimental environment, proceeds to validate the effectiveness of ADM-DDA6 through experiments, and concludes by testing its real-time detection performance overhead under various loads.

### Experimental setup

This paper employs threat vectors from two tools. The first tool is THC-IPv6^49^, utilizing the denial6 and fragmentation6 tools to generate threat packets based on IPv6 extension headers. The second tool is ExtHdr, developed by this paper using Chiron^22^ and Scapy^50^. Drawing on an investigation into the prior work of IPv6 extension header threats, this paper identified and implemented 40 common extension header threats through ExtHdr.

**Figure 7:**
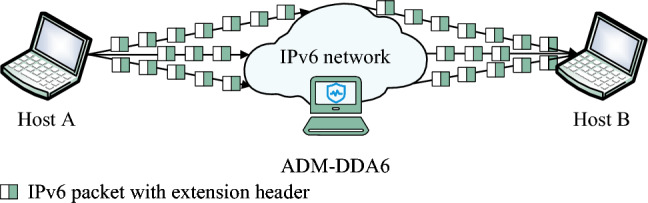
Topology of IPv6 network experimental environment.

The experimental setup depicted in Fig. 7 is established in this paper. Host A runs Ubuntu 20.04 (kernel version: Linux 5.15), equipped with an AMD Ryzen 9 5900HX CPU@ 3.30GHz and 8GB of memory. THC-IPv6 and ExtHdr tools are installed on Host A. Host B runs Ubuntu 23.04 (kernel version: Linux 6.2), equipped with an AMD Ryzen 9 5900HX CPU@ 3.30GHz and 8GB of memory. Host B is configured with Suricata v6.0.12, Snort v3.1.61.0, and ADM-DDA6.

### Comparison of threat detection types

To validate the effectiveness of the proposed standard rule sets in this paper, a comparative analysis of threat detection capabilities for different methods was conducted using both the THC-IPv6 and ExtHdr tools.

The compared methods include Suricata v6.0.12, Snort v3.1.61.0, and ADM-DDA6. To eliminate the influence of other rules, Suricata and Snort were configured with only default rule sets related to IPv6. Suricata’s default rule set is ET/OPEN, containing approximately 34 rules related to IPv6 extension header threats, while Snort has 26 rules. ADM-DDA6 uses 20 rules.

**Figure 8:**
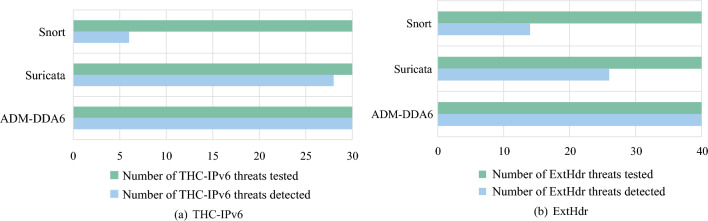
Detection types for two tools THC-IPv6 and ExtHdr.

The experimental results, shown in Fig. 8a,b, indicate that when experiments are conducted using the THC-IPv6 tool, Suricata detects 28 threats, while Snort detects only 6. In experiments with the ExtHdr tool, Snort and Suricata detected 14 and 26 threats, respectively. In contrast, ADM-DDA6 successfully detected all 70 threat types from both tools, demonstrating a substantial improvement in threat detection capabilities.

It is worth noting that, in response to the IPv6 fragment evasion threat outlined in Section “Formulating rules based on RFC 7112 (An example of IPv6 fragment evasion threats)” , ExtHdr has developed two variations: IPv6 fragment evasion threat with known and unknown upper-layer protocols^29,40^, respectively. According to experimental results, it is evident that ADM-DDA6 can detect both threat types.

Suricata and Snort exhibit a constrained ability to detect a limited range of threat types associated with IPv6 extension headers due to the finite number of rules. Specifically, in this experiment, Suricata utilizes 34 rules, surpassing the 20 standard rules proposed in ADM-DDA6. Despite the larger rule set, Suricata’s rules are insufficient to cover all the known threat types related to IPv6 extension headers. This emphasizes the effectiveness of the standard rules presented in ADM-DDA6, achieving comprehensive threat coverage with a more compact set of rules.

The experimental results reveal Snort’s subpar performance in detecting IPv6 extension header threats. Suricata displayed inconsistent detection rates when faced with threats generated by the two tools. Specifically, with the THC-IPv6 tool, Suricata exhibits a higher detection rate, whereas with the ExtHdr tool, its detection rate is relatively lower.

**Figure 9:**
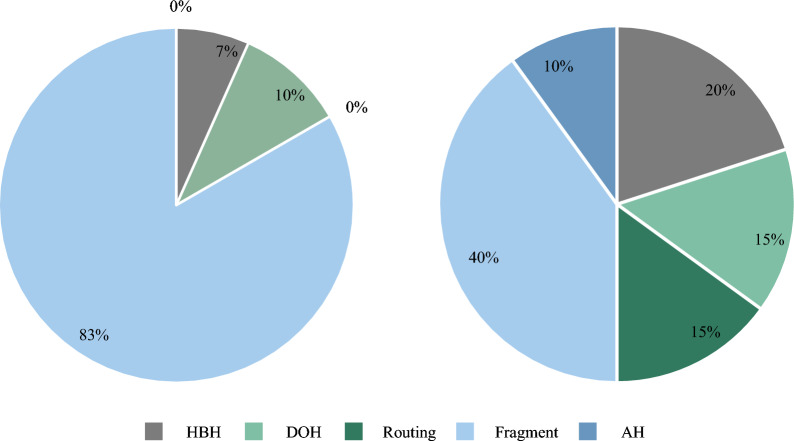
Threat distribution of the two tools THC-IPv6 and ExtHdr: THC-IPv6 on the left and ExtHdr on the right.

To understand the underlying reasons for this distinction, this paper conducts a detailed analysis of the threat types generated by both the THC-IPv6 tool and the ExtHdr tool. As illustrated in Fig. 9, threats linked to the Fragment headers in THC-IPv6 tool constitute the majority, reaching 83%, with threats related to the HBH and DOH headers being the least prevalent. However, threats associated with other header types are absent. Conversely, the distribution of threat types from the ExtHdr tool is more evenly spread across various IPv6 extension header types.

In both tools, threats linked to the Fragment header exhibit a higher proportion. This is attributed to the various types of threats associated with Fragment headers, including overlapping fragments, atomic fragments, nested fragments, fragment evasion threats, tiny fragments, and more^51^. Accordingly, this paper formulates 8 rules specifically for Fragment headers, as outlined in Table 2. Considering that threats generated by the ExtHdr tool are more evenly distributed across different extension header types, we opt to concentrate on research based on ExtHdr in subsequent experiments.

### Proof of universality

The ADM-DDA6 model is designed to be implementable in any language or environment, free from dependencies on specific software or hardware. While Section “Running example verification” demonstrates the effectiveness of using this model to parse IPv6 extension header packets through examples, it is essential to assess the generality of ADM-DDA6 when confronted with diverse and complex IPv6 extension header packets.

To further verify the performance of ADM-DDA6 in more complex environments, this paper designs and implements more detailed and extensive experiments based on Table 3, which is manifested by increasing the number and types of IPv6 extension headers. The results, as shown in Table 4, show that ADM-DDA6 can still detect anomalies and issue warnings on time under these complex test conditions.

**Table 4:** The universality test of ADM-DDA6.

IPv6 header chain types	IPv6 header chain styles	ADM-DDA6 behaviors
Normal	A+B+C+D+E	**Normal**
Header disorder	A+C+B+D+E+More IPv6 extension headers	**Abnormal**
	A+B+D+C+E+More IPv6 extension headers	**Abnormal**
	A+D+E+B+C+More IPv6 extension headers	**Abnormal**
Header repetition	A+B+B+C+D+E+More IPv6 extension headers	**Abnormal**
	A+B+C+C+D+E+More IPv6 extension headers	**Abnormal**
	A+B+C+D+D+E+More IPv6 extension headers	**Abnormal**
Header disorder + repetition	A+C+B+B+D+E+More IPv6 extension headers	**Abnormal**
Header repetition + disorder	A+B+B+D+C+E+More IPv6 extension headers	**Abnormal**

Significant values are in bold.

The reasons are as follows, ADM-DDA6 immediately stops parsing and triggers the warning mechanism when it recognizes the first anomaly (including, but not limited to, header duplication or disorder), regardless of the number of consecutive IPv6 extension headers in the upper layer protocol. Second, 6 types of IPv6 extension headers are considered in the experiment. Therefore, before encountering the first anomaly, even when facing packets containing multiple types of massively extended headers, ADM-DDA6 only needs to check at most the first 6 consecutive IPv6 extension headers to ensure timely response and accurate judgment of the anomaly, thus saving system resources.

In fact, the threats provided by the THC-IPv6 and ExtHdr tools in the experiments from Section “Comparison of threat detection types” inherently contain complex and diverse IPv6 extension header packets. This also substantiates the universality of ADM-DDA6.

### Comparison of threat detection time

**Figure 10:**
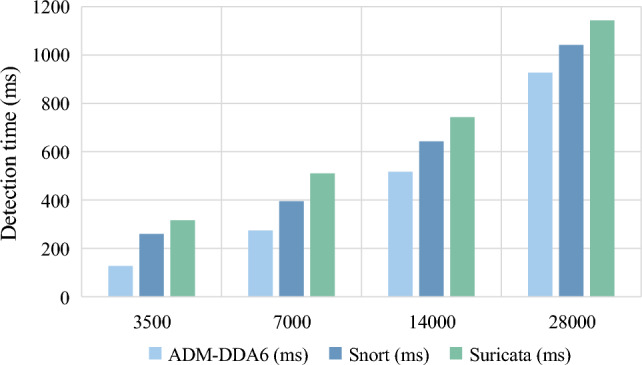
Comparison of detection time under different numbers of threats.

Using the ExtHdr tool, 3500, 7000, 14000, and 28000 threat packets were generated, and the time costs for threat detection by Snort, Suricata, and ADM-DDA6 for different quantities of packets were tested. The results are shown in Fig. 10.

Observing the result, it is evident that as the number of packets increases, the required parsing time for Snort, Suricata, and the proposed method all show an increasing trend. It is noticeable that ADM-DDA6 requires less parsing time compared to Snort and Suricata, with detection speeds improved by 21.2% and 32%, respectively.

The improvement in detection speed can be attributed to the following factors:

- ADM-DDA6 employs adaptive rule matching during the threat detection process. Taking the packet in Fig. 1 as an example, traditional IDS would require numerous rule matches, resulting in the identification of 3 rules and the issuance of 3 warnings. In contrast, ADM-DDA6, when matching rules for the DOH header, detects the anomaly and promptly transitions to the abnormal final state, eliminating the need to parse subsequent headers.
- This paper formulates standard RFC-compliant rule sets, resulting in a significantly lower number of rules for ADM-DDA6 (20 rules) compared to Suricata (34 rules). The reduced number of rules also translates to diminished time overhead.

In summary, regardless of the quantity of threat packets, ADM-DDA6 consistently demonstrates a lower detection time than Snort and Suricata. This affirms the effective enhancement of detection speed by ADM-DDA6.

### Detection performance and overhead Analysis

To assess the detection performance overhead of ADM-DDA6, this section conducts tests from two perspectives: operational resource overhead and impact on network performance.

#### Running overhead of ADM-DDA6

Due to the lack of relevant research on detection performance overhead in the current field, this section opts for a longitudinal comparison, i.e., evaluating the change in resource overhead of ADM-DDA6 in scenarios without threats (Scenario 1) and with threats encountered (Scenario 2), to gain a deeper understanding of the model’s performance.

Firstly, to evaluate the impact of different packet time intervals on the detection accuracy of ADM-DDA6, a series of test cases were sent using the ExtHdr tool with varying packet time intervals.

This paper conducts 4 rounds of experiments, gradually shortening the packet intervals to simulate the detection accuracy of ADM-DDA6 at different packet transmission rates and determine the performance limits of the verification method under the minimum packet interval. The experimental results are shown in Table 5.

**Table 5:** Detection accuracies of different packet time intervals.

Time intervals	10 ms	5 ms	2 ms	1.5 ms	1 ms	0.8 ms	0.6 ms	0.5 ms
Experiment 1	**100%**	**100%**	**100%**	99.11%	98.40%	97.43%	95.15%	90.33%
Experiment 2	**100%**	**100%**	**100%**	99.22%	98.47%	97.60%	95.20%	90.28%
Experiment 3	**100%**	**100%**	**100%**	99.30%	99.00%	97.60%	95.25%	90.92%
Experiment 4	**100%**	**100%**	**100%**	99.35%	98.73%	98.00%	95.85%	91.40%

Significant values are in bold.

The results indicate that when the packet time interval is 2 ms (transmission rate of 500 pps), ADM-DDA6 achieves a detection rate of 100%. As the packet transmission rate increases (i.e., the packet time interval decreases), the detection accuracy of ADM-DDA6 for all types of threats gradually decreases. When the packet time interval is below 0.6 ms (transmission rate of 1667 pps), the detection rate is noticeably below 95%. Therefore, it can be concluded that the minimum packet interval acceptable for ADM-DDA6 is 2 ms.

Next, this paper tests the performance overhead required by ADM-DDA6. Using the sysstat tool, the system performance of the proposed method was evaluated in two different scenarios^52^, namely, Scenario 1 and Scenario 2. In this experiment, various test cases were sent to the destination host at a 2ms interval, and the experiment was conducted for 100 rounds. The evaluation included changes in CPU (Central Processing Unit) performance and memory performance.

**Figure 11:**
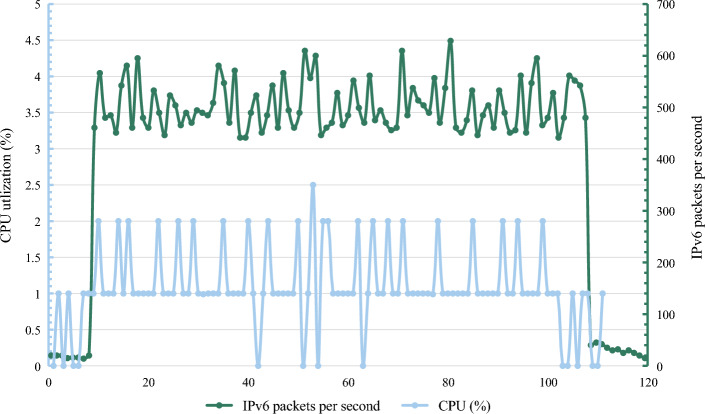
Running overhead of ADM-DDA6.

Figure 11 depicts the quantity of IPv6 packets the destination host received over time and the corresponding CPU utilization. As observed, a substantial number of test cases, corresponding to Scenario 2, were received between 10 seconds and 100 seconds, while the rest of the time fell under Scenario 1.

Notably, ADM-DDA6 did not exhibit a significant increase in CPU utilization during Scenario 2 compared to normal conditions. This suggests that ADM-DDA6 incurs relatively low-performance overhead in real-world environments, even when facing large-scale threat detection, with no significant elevation in CPU usage.

Regarding changes in memory performance, the experimental results indicate a consistent memory overhead of 0.53% in both scenarios. This implies that there was no noticeable increase in memory performance under the given conditions.

#### The impact of ADM-DDA6 on network performance

To evaluate the actual influence of ADM-DDA6 on network performance, this study devised the following experimental setup based on the topology depicted in Fig. 7: Host A continuously transmits threat packets to Host B. Host B is divided into two scenarios: one with ADM-DDA6 deployed and the other without ADM-DDA6 deployment.

Additionally, this study employs a third host to send ICMPv6 Echo Request messages to Host B, measuring the RTT (Round-Trip Time) and packet loss rate between the request and response messages. RTT reflects the network latency, with higher RTT values indicating degraded network performance. The packet loss rate, on the other hand, signifies the reliability of network data transmission. An increase in packet loss rate directly impacts communication quality, thereby affecting the normal operation of programs.

By comparing the changes in RTT and packet loss rate between the scenarios with and without ADM-DDA6 deployment, this research aims to provide a clear demonstration of the specific impact of ADM-DDA6 on network performance.

**Figure 12:**
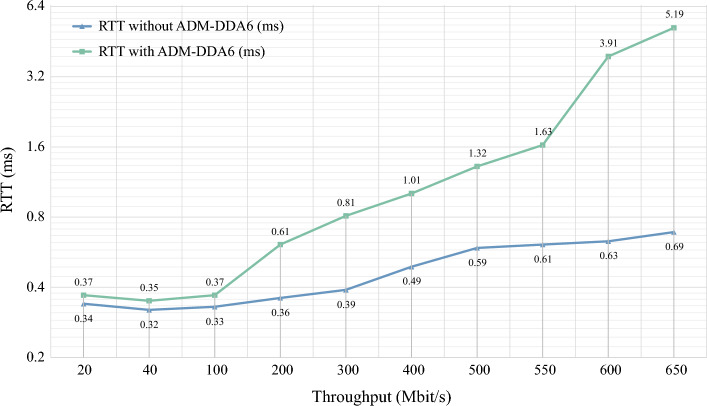
The impact of ADM-DDA6 on RTT.

The impact of ADM-DDA6 on RTT is illustrated in Fig. 12, while the impact on packet loss rate is presented in Table 6. With an increase in throughput, both scenarios, with and without ADM-DDA6, show a rising trend in RTT. At lower throughputs (0–100 Mbit/s), ADM-DDA6 exhibits minimal impact on RTT, with an increase of approximately 0.03ms and a packet loss rate of 0 for both scenarios. In the moderate throughput range (200–500 Mbit/s), ADM-DDA6 leads to a more noticeable increase in RTT, with an increment of about 0.48 ms, while the packet loss rate remains at 0 for both scenarios. However, when the throughput exceeds 500 Mbit/s, the impact of ADM-DDA6 on RTT becomes more pronounced, with an increase of approximately 2.93 ms, accompanied by a noticeable rise in the packet loss rate.

**Table 6:** The impact of ADM-DDA6 on packet loss rate.

Throughput (Mbit/s)	20	40	100	200	300	400	500	550	600	650
Packet loss rate without ADM-DDA6	0%	0%	0%	0%	0%	0%	0%	0%	0%	0%
Packet loss rate with ADM-DDA6	0%	0%	0%	0%	0%	0%	0%	**10.5%**	**21.31%**	**51.12%**

Significant values are in bold.

Notably, under the experimental conditions of this study, the network’s maximum throughput steady at 650 Mbit/s, regardless of the implementation of the ADM-DDA6 method, indicating its negligible effect on throughput.

Overall, ADM-DDA6 does not affect the maximum throughput of the network. Its influence on network performance is minimal at lower or moderate throughputs, with the increase in RTT within acceptable limits and no observed packet loss. However, under conditions of higher throughput, ADM-DDA6 may lead to a significant increase in RTT and a rise in packet loss rate, potentially adversely affecting network performance.

### Comparison with the detection capability of existing methods and scalability analysis

Since this study compares the resource overhead of ADM-DDA6 when threats are present versus absent, as well as the impact on network performance with and without ADM-DDA6, without comparing it to other security solutions, this section will focus solely on comparing detection types, detection universality, and time cost.

Table 7 presents a comparative analysis between ADM-DDA6 and several existing security methods, also contrasting with current mainstream open-source IDSs.

**Table 7:** Comparison with existing security methods.

Detecting methods	Detection types	Detection universality	Time cost
Snort^32^	14	Yes	Middle
Suricata^31^	26	Yes	High
Ofsoftswitch^29^	2	No	–
DEH-DoSv6^30^	24	No	–
NOPO^28^	14	No	–
Suricata6+	40	Yes	High
**ADM-DDA6**	**40**	**Yes**	**Low**

Significant values are in bold.

In terms of threat detection types, Snort and Suricata can identify 14 and 26 threats, respectively. This limitation stems from the finite threat types covered in their rule sets for IPv6 extension header threats. Ofsoftswitch, DEH-DoSv6 (a defendable security model against IPv6 extension headers denial of service attack), and NOPO employ DPI-based detection methods, each capable of identifying a limited set of threats. Ofsoftswitch can only detect 2 types of fragment evasion threats. DEH-DoSv6 can identify 24 threats, with 16 of them related to fragment header threats. In contrast, ADM-DDA6 can detect 40 threats generated by the ExtHdr tool.

In terms of universality, Snort, and Suricata, being signature-based IDS, inherently possess generality. In contrast, Ofsoftswitch, DEH-DoSv6, and NOPO lack generality. Ofsoftswitch relies on OpenFlow 1.3.0 support for IPv6 extension header threat detection and integrates with OpenFlow flow entries for packet forwarding control, restricting its operation to OpenFlow networks. DEH-DoSv6 is a method dependent on router devices, while NOPO can only parse a limited set of IPv6 extension headers. In comparison, ADM-DDA6 proposes a threat detection model based on DFA theory, capable of parsing complex and variable IPv6 extension header packets, exhibiting excellent universality.

In terms of time cost, as depicted in Fig. 10, it is evident that Suricata incurs the highest time cost across different threat magnitudes, followed by Snort, while ADM-DDA6 exhibits the lowest. The primary reason is the abundance of rules in Suricata, leading to potentially multiple unnecessary matches during the rule matching process, thereby consuming more time. In contrast, ADM-DDA6 achieves lower time costs due to the utilization of the adaptive rule matching method, reducing the time required for rule matching. Additionally, the method of rule formulation based on RFC specifications results in a lower rule quantity, further diminishing the time needed for rule matching.

To investigate the scalability of ADM-DDA6, this study initially integrated the main concepts of ADM-DDA6 into Suricata, resulting in Suricata6+, which was then subjected to testing. As shown in Table 7, Suricata6+ successfully detected 40 threats constructed by the ExtHdr tool and identified complex and variable combinations of IPv6 header chains. However, its time cost remained similar to that of Suricata, still relatively high.

Upon analysis, it is evident that the improvements made to Suricata were limited to rule formulation for IPv6 extension headers, header parsing, and threat detection. Handling other types of threats and rule formulation were not the primary focus of this study.

Consequently, Suricata6+ still requires parsing and matching of a greater variety of rules compared to ADM-DDA6, necessitating additional processing. For instance, Suricata6+ utilizes a total of 41,658 rules (i.e., Suricata: 41,638, ADM-DDA6: 20). The process of parsing rules alone consumes a significant amount of resources during threat packet detection and rule matching, resulting in higher time cost. However, the time cost of Suricata6+ did not significantly increase compared to Suricata, indicating the effectiveness of the ADM-DDA6 approach.

## Conclusion

This study addresses key issues in detecting threats posed by IPv6 extension headers, namely deficiencies in detection variety, detection universality, and detection speed, observed in existing solutions. In this context, we innovatively adapt and expand rule matching methods to propose an adaptive detection model for IPv6 extension header threats, termed ADM-DDA6, based on DFA theory.

This method offers several advantages: Construction of standardized rule sets for IPv6 extension headers based on RFC specifications, reducing redundant rules and broadening the scope of threat detection types; Application of DFA theory to the parsing and detection process of IPv6 extension header packets, effectively addressing the challenges posed by complex and flexible header combinations, thereby enhancing adaptability to intricate header structures; Introduction of an adaptive rule matching method, wherein only the rule set corresponding to the current header is matched during header parsing, enabling rapid termination of detection upon detecting anomalies and thus improving detection efficiency.

While the ADM-DDA6 method has demonstrated significant efficacy in detecting threats associated with IPv6 extension headers, it also presents certain limitations in practical applications. Firstly, while effective for detecting threats with static behavioral characteristics, the rule-based approach may not adequately address all threats exhibiting dynamic behaviors. Secondly, in the experimental environment of this study, ADM-DDA6 demonstrated minimal impact on network performance at lower or moderate network throughputs but exhibited some influence under conditions of higher throughput.

In future work, efforts will be directed towards addressing these limitations. Optimization of high-performance hardware infrastructure will be pursued to enhance overall network throughput. Furthermore, validation of the threat detection efficacy of the ADM-DDA6 model will be conducted in high-speed network environments closer to real-world scenarios.

## Data Availability

The data that support the findings of this study are available from the corresponding author, upon reasonable request.
